# Gender difference in determinant factors of being overweight among the 40–70-year-old population of Kharameh cohort study, Iran

**DOI:** 10.1186/s12889-021-10802-z

**Published:** 2021-04-17

**Authors:** Seyed Alireza Dastgheib, Abbas Rezaianzadeh, Najmeh Maharlouei, Salar Rahimikazerooni, Kamran B. Lankarani

**Affiliations:** 1 Student Research Committee, Shiraz University of Medical Sciences, Shiraz, Iran; 2 Colorectal Research Center, Shiraz University of Medical Sciences, Shiraz, Iran; 3 Health Policy Research Center, Institute of Health, Shiraz University of Medical Sciences, Shiraz, Iran

**Keywords:** Overweight, Gender difference, Socio-demographic factors, Iran

## Abstract

**Background:**

Iranians face being overweight as one of the most common health problems, which is more prevalent among women. This study aimed to identify gender differences in determinants of being overweight in 40- to 70-year-old participants from Kharameh, Iran.

**Methods:**

This cross-sectional study was conducted during 2015–2016. The total 10,663 inhabitants of Kharameh, Iran, aged 40–70 years old, were target population. Those with a body mass index (BMI) < 18.5 or > 29.9 were excluded. A checklist composed of socio-demographic, lifestyle, and BMI items was used; a *p*-value < 0.05 was considered significant.

**Results:**

Overall, 53.4% of 8222 participants were overweight. The prevalence of overweight women (62.7%) was significantly higher (*p* <  0.001) than men (43.6%).

The logistic regression model for men showed that being overweight was more likely among men with cigarette smoking history (OR = 1.49) and those with a moderate physical activity level (OR = 1.35), but less likely among those with a higher socio-economic status (SES) (OR = 0.74). Among women, being overweight was associated with high SES (OR = 1.61), an education level below high school diploma (OR = 1.57) and primary school education (OR = 1.50), being married (OR = 2.39), widowed (OR = 2.11) and having a greater calorie intake (OR = 1.01). Being overweight was less likely among employed women (OR = 0.85), those with cigarette smoking history (OR = 0.65), and those with high (OR = 0.72) and intensive physical activity (OR = 0.73).

**Conclusions:**

This study revealed the gender differences in determining factors affecting being overweight. As being overweight was more prevalent among women, the priority of health policies to control this issue should also be focused on women.

## Background

High body mass index (BMI) is one of the most common health problems in both developed and developing countries [1]. It is one of the main reasons for morbidity, mortality, and even impaired quality of life [2]. Worldwide, being overweight is increasing [3]. According to the World Health Organization (WHO), more than 1.9 billion adults were overweight in 2016 [4].

Iran, like other countries, is facing the challenge of increasing number of overweight individuals. Based on a systematic review with data from January 2005 to January 2014, the range of overweight individuals among the Iranian adult population at the sub-national level was between 12.8 and 76.4% [5]. However, studies after 2014 in Iran have shown rates of being overweight to be 39.6% in East Azerbaijan [6], 36.5% in West Azerbaijan [7], between 34.1 and 63.6% among those aged above 20 years in Tehran [1, 8], and 43.4% among 40–64-year-old people in Shahroud [9].

The existing literature shows associations between BMI and some socio-demographic characteristics such as age, gender, marital status, race, educational level, occupational status, and socio-economic status (SES) [1, 6, 10–12]. However, there has been some controversial results regarding some of the associations, such as SES and BMI [9, 13, 14]. In addition, studies have identified low level of physical activity as a significant risk factor for being overweight [15, 16]. Smoking status has also been found to be inversely associated with BMI [11].

However, there are differences in determining factors among genders [17]. The prevalence of overweight individuals is higher among women [1, 6, 9, 12]. Studies have shown that married, unemployed, and low-educated women were at a higher risk of being overweight [1, 11, 12, 17]. Moreover, smoking status was associated with higher weight only in women [17]. On the other hand, another study presented different findings, showing that among both genders, nonsmokers were more expected to be overweight or obese [6].

The current study aimed to identify the association between socio-demographic and lifestyle factors and being overweight in the 40–70–year-old population of Kharameh city. As the prevalence of being overweight was higher among women [6, 9, 12], determinants associated with being overweight that differed among men and women were also investigated.

## Methods

### Study design and participants

This analytical cross-sectional study was conducted using the baseline data of the Kharameh cohort study, a branch of the Prospective Epidemiological Research Studies in Iran (PERSIAN cohort). The rationale, objectives, and design of the PERSIAN cohort study have earlier been published [18, 19]. This study was approved by Shiraz University of Medical Sciences Ethics Committee (IR.sums.med.rec.1398.340).

Kharameh is one of the counties of Fars province, the fifth populous province located in southwestern Iran. According to the latest national census in 2016, its population was 54,864, 14,447 of whom were 40 to 70 years old [20]. Out of 22,939 participants in the Fars cohort [18], 8222 individuals were aged 40 to 70 years old, and all of them were entered into the study. The inclusion criteria were living in Kharameh for at least 1 year before the start of this study, and willingness to participate. Participants with a BMI below 18.5 or greater than 29.99 were excluded.

### Data gathering tools

Data was collected with a checklist consisting of three main parts: socio-demographic characteristics, lifestyle variables, and anthropometric indices. Socio-demographic variables were comprised of age, gender, marital status (married, single, widowed/divorced), educational level (illiterate, under diploma, high school diploma, university degree), occupational status (employed, unemployed), and SES (low, lower-middle, upper-middle, and high). The SES of households was calculated using the principal component analysis method and included assets of the participants such as type of residence (owned or rented), residential area (in square meters), number of rooms, ownership of landline telephone, washing machine, dishwasher, flat-screen TV, refrigerator, vacuum cleaner, or personal computer/laptop, access to Internet at home, access to a shower and toilet, and car ownership status and its value.

Lifestyle variables included history of cigarette smoking (yes-no), history of hookah smoking (yes-no), history of alcohol drinking (yes-no), level of physical activity (light, moderate, high, and intensive), and calorie intake. Participants’ daily physical activity was measured through metabolic equivalent rates (METs) using a self-reported validated questionnaire. One MET is equal to resting metabolic rate, the amount of oxygen consumed at rest which is about 3.5 ml 02/kg/min. As four METs require 16 ml 02/kg/min, the MET for each activity was extracted using a compendium of physical activities [18]. To measure calorie intake, the food frequency questionnaire (FFQ) was used. It contains 130 food items [19], and has been validated for Iranian populations based on their culture and food habits [21]. Participant BMIs were calculated as weight in kilograms divided by square of height in meters. According to WHO, BMI is considered normal if it is between 18.5 and 25 and overweight if it is between 25.0 and 30 [22]. The dependent variable used in this study was the classification variable (0 for those with normal weight, 1 for overweight participants).

### Data analysis

The Statistical Package for Social Sciences (SPSS) software version 21 (IBM, Armonk, NY, USA) was used for data analysis. Descriptive analysis including mean, standard deviation, and frequency distribution was done to assess demographic and anthropometric characteristics. Chi-square test was used as a univariate analysis for associations between the independent variables and the outcome variable. A binary logistic regression analysis was applied to determine the predictive variables of being overweight among 40- to 70-year-old adults in Kharameh. According to the univariate analyses, variables with *p* ≤ 0.2 had the necessary requirement to be entered into the regression model. The final model was reported with odds ratios (ORs) and 95% confidence intervals (CIs). A *p*-value less than 0.05 was considered significant.

## Results

Overall, 8222 adults were studied, out of which 51.4% were women. The mean BMI for the total population was 24.99 ± 2.9 kg/m^2^, 24.34 ± 2.9 kg/m^2^ for men and 25.62 ± 2.8 kg/m^2^ for women. The prevalence of being overweight was 53.4% and higher among women (62.7%) than men (43.6%). Other anthropometric information is shown in Table 1.

**Table 1:** Anthropometric indices in the studied sample according to gender

Anthropometric Variable	Total	Gender	
		Men	Women
Number of subjects	8222 (100.0)	3992 (48.6)	4230 (51.4)
Height (cm)	163.65 (± 9.6)	170.79 (± 7.1)	156.91 (± 6.3)
Weight (kg)	67.05 (± 10.2)	71.14 (± 10.3)	63.20 (± 8.5)
BMI (kg/m^2^)	24.99 (± 2.92)	24.34 (± 2.9)	25.62 (± 2.8)
Normal weight (18.5 **≤** BMI **<** 25.0)	3828 (46.6)	2249 (56.4)	1579 (37.3)
Overweight (25.0 **≤** BMI **<** 30.0)	4390 (53.4)	1741 (43.6)	2649 (62.7)

Data is reported as mean (± SD) or frequency (%) as indicated

Regardless of gender, the variables of age, SES, employment status, marital status, level of education, physical activity, and history of alcohol drinking and cigarette smoking, differed significantly (*p* <  0.001) between overweight individuals and those with normal weight (Table 2).

**Table 2:** Association between individual characteristics and normal weight and overweight among studied population

Variable	Gender	Sub-group	Number of subjects (%)	Normal weight (%)	Overweight (%)
Age group	Men	40–49	1988 (49.8)	1148 (51.1)	840 (48.3)
		50–59	1441 (36.1)	791 (35.2)	648 (37.3)
		≥60	559 (14.0)	309 (13.7)	250 (14.4)
		^1^*p*-value		0.229	
	Women	40–49	1659 (39.2)	632 (40.1)	1026 (38.7)
		50–59	1504 (35.6)	563 (35.7)	940 (35.5)
		≥60	1066 (25.2)	383 (24.3)	683 (25.8)
		^1^*p*-value		0.51	
	Total	40–49	3647 (44.4)	1780 (46.5)	1866 (42.5)
		50–59	2945 (35.8)	1354 (35.4)	1588 (36.2)
		≥60	1625 (19.8)	692 (18.1)	933 (21.3)
		^1^*p*-value		< 0.001	
SES	Men	Low	801 (20.1)	522 (23.2)	279 (16.0)
		Lower-middle	874 (21.9)	535 (23.8)	338 (19.4)
		Upper-middle	840 (21.0)	474 (21.1)	366 (21.0)
		High	1477 (37.0)	718 (31.9)	758 (43.5)
		^1^*p*-value		< 0.001	
	Women	Low	1261 (29.8)	535 (33.9)	725 (27.4)
		Lower-middle	1383 (32.7)	531 (33.6)	852 (32.2)
		Upper-middle	1078 (25.5)	373 (23.6)	704 (26.6)
		High	508 (12.0)	140 (8.9)	368 (13.9)
		^1^*p*-value		< 0.001	
	Total	Low	2062 (25.1)	1057 (27.6)	1004 (22.9)
		Lower-middle	2257 (27.5)	1066 (27.8)	1190 (27.1)
		Upper-middle	1918 (23.3)	847 (22.1)	1070 (24.4)
		High	1985 (24.1)	858 (22.4)	1126 (25.6)
		^1^*p*-value		< 0.001	
Employment status	Men	Employed	3402 (85.2)	1925 (85.6)	1477 (84.8)
		Unemployed	590 (14.8)	324 (14.4)	264 (15.2)
		^1^*P-value*		0.266	
	Women	Employed	1141 (27.0)	472 (29.9)	1980 (74.7)
		Unemployed	3089 (73.0)	1107 (70.1)	669 (25.3)
		^1^*p*-value		0.001	
	Total	Employed	4543 (55.3)	2397 (62.6)	2146 (48.9)
		Unemployed	3679 (44.7)	1431 (37.4)	2244 (51.1)
		^1^*p*-value		< 0.001	
Marital status	Men	Single	21 (0.5)	11 (0.5)	9 (0.5)
		Married	3940 (98.7)	2217 (98.6)	1722 (98.9)
		Widowed/divorced	31 (0.8)	21 (0.9)	10 (0.6)
		^1^*p*-value		0.437	
	Women	Single	116 (2.7)	63 (4.0)	53 (2.0)
		Married	3445 (81.4)	1241 (78.6)	2202 (83.1)
		Widowed/divorced	669 (15.8)	275 (17.4)	394 (14.9)
		^1^*p*-value		< 0.001	
	Total	Single	137 (1.7)	74 (1.9)	62 (1.4)
		Married	7385 (89.8)	3458 (90.3)	3924 (89.4)
		Widowed/divorced	700 (8.5)	296 (7.7)	404 (9.2)
		^1^*p*-value		0.013	
Education level	Men	Illiterate	843 (21.1)	515 (22.9)	328 (18.8)
		Under diploma	1610 (40.3)	915 (40.7)	694 (39.9)
		high school diploma	1178 (29.5)	651 (28.9)	526 (30.2)
		University degree	361 (9.0)	168 (7.5)	193 (11.1)
		^1^*P-value*		< 0.001	
	Women	Illiterate	1747 (41.3)	767 (48.6)	979 (37.0)
		Under diploma	1865 (44.1)	619 (39.2)	1246 (47.0)
		high school diploma	494 (11.7)	153 (9.7)	340 (12.8)
		University degree	124 (2.9)	40 (2.5)	84 (3.2)
		^1^*p*-value		< 0.001	
	Total	Illiterate	2590 (31.5)	1282 (33.5)	1307 (29.8)
		Under diploma	3475 (42.3)	1534 (40.1)	1940 (44.2)
		high school diploma	1672 (20.3)	804 (21.0)	866 (19.7)
		University degree	485 (5.9)	208 (5.4)	277 (6.3)
		^1^*p*-value		< 0.001	
Physical activity	Men	Light	1178 (29.5)	617 (27.4)	559 (32.1)
		Moderate	650 (16.3)	322 (14.3)	328 (18.8)
		High	577 (14.5)	314 (14.0)	263 (15.1)
		Intensive	1587 (39.8)	996 (44.3)	591 (33.9)
		^1^*p*-value		< 0.001	
	Women	Light	836 (19.8)	286 (18.1)	549 (20.7)
		Moderate	1349 (31.9)	480 (30.4)	868 (32.8)
		High	1444 (34.1)	571 (36.2)	873 (33.0)
		Intensive	601 (14.2)	242 (15.3)	359 (13.6)
		^1^*p*-value		0.016	
	Total	Light	2014 (24.5))	903 (23.6)	1108 (25.2)
		Moderate	1999 (24.3)	802 (21.0)	1196 (27.2)
		High	2021 (24.6)	885 (23.1)	1136 (25.9)
		Intensive	2188 (26.6)	1238 (32.3)	950 (21.6)
		^1^*p*-value		< 0.001	
History of alcohol drinking	Men	Yes	458 (11.5)	266 (11.8)	191 (11.0)
		No	3534 (88.5)	1983 (88.2)	1550 (89.0)
		^1^*p*-value		0.214	
	Women	Yes	6 (0.1)	3 (0.2)	3 (0.1)
		No	4224 (99.9)	1576 (99.8)	2646 (99.9)
		^1^*p-value*		0.401	
	Total	Yes	464 (5.6)	269 (7.0)	194 (4.4)
		No	7758 (94.4)	3559 (93.0)	4196 (95.6)
		^1^*P-value*		< 0.001	
History of cigarette smoking	Men	Yes	2067 (51.8)	1277 (56.8)	789 (45.3)
		No	1925 (48.2)	972 (43.2)	952 (54.7)
		*p-value*		< 0.001	
	Women	Yes	146 (3.5)	74 (4.7)	72 (2.7)
		No	4084 (96.5)	1505 (95.3)	2577 (97.3)
		^1^*p*-value		0.001	
	Total	Yes	2213 (26.9)	1351 (35.3)	861 (19.6)
		No	6009 (73.1)	2477 (64.7)	3529 (80.4)
		^1^*p*-value		< 0.001	
History of hookah smoking	Men	Yes	384 (9.6)	193 (8.6)	190 (10.9)
		No	3608 (90.4)	2056 (91.4)	1551 (89.1)
		^1^*p*-value		0.008	
	Women	Yes	58 (1.4)	22 (1.4)	35 (1.3)
		No	4172 (98.6)	1557 (98.6)	2614 (98.7)
		*p*-value		0.472	
	Total	Yes	442 (5.4)	215 (5.6)	225 (5.1)
		No	7780 (94.6)	3613 (94.4)	4165 (94.9)
		^1^*p*-value		0.324	
Calorie intake*	Men	–	3431.00 ± 1087.81	3421.85 ± 1072.11	3424.06 ± 1106.64
		^2^*p*-value		0.815	
	Women		3126.73 ± 1002.43	3053.18 ± 980.16	3148.93 ± 1011.13
		^2^*p*-value		0.003	
	Total		3261.38 ± 1051.94	3269.76 ± 1050.84	3257.52 ± 1058.32
		^2^*p*-value		0.599	

All data is reported as frequency (%), except calorie intake which is reported as Mean (±Standard Deviation)

^1^Chi-square test was used

^2^T test was used

BMI was also statistically different between men and women (*p* <  0.001). Among the studied variables, SES (*p* <  0.001), education level (*p* <  0.001), physical activity (*p* <  0.001), history of cigarette smoking (*p* <  0.001), and history of hookah smoking (*p* = 0.008) were significantly different between overweight men and those with a normal weight (Table 2).

For women, SES (*p* <  0.001), employment status (*p* = 0.001), marital status (*p* <  0.001), education level (*p* <  0.001), physical activity (*p* = 0.016), history of cigarette smoking (*p* = 0.001), and calorie intake (*p* = 0.003) were significantly different between overweight women and those with a normal weight (Table 2).

Results from logistic regression to predict factors associated with being overweight in the total population showed that women (OR = 2.002), those with upper-middle and high SES (OR = 1.277 and 1.684, respectively), married (OR = 1.975) and widowed/divorced people (OR = 1.699), and among individuals with any level of education up to primary school education (*p* <  0.05) were more likely to be overweight. However, people with a history of cigarette smoking (OR = 0.630, *p* <  0.001), with high (OR = 0.802) and intensive physical activity levels (OR = 0.720, *p* <  0.001) were less likely to be overweight (Table 3).

**Table 3:** Regression model indicating factors associated with overweight in total population

Variables retained in model	Odd’s Ratio (OR)	95% Confidence Interval for OR	*p*-value
Age			
40–49	1		
50–59	1.095	(0.989–1.213)	0.080
≥60	1.123	(0.992–1.272)	0.066
SES			
Low	1		
Lower-middle	1.119	(0.988–1.268)	0.076
Upper-middle	1.277	(1.119–1.458)	< 0.001
High	1.684	(1.458–1.945)	< 0.001
Gender			
Male	1		
Female	2.002	(1.744–2.299)	< 0.001
Education level			
Illiterate	1		
Up to primary school	1.365	(1.224–1.522)	< 0.001
Under diploma	1.343	(1.166–1.546)	< 0.001
University degree	1.317	(1.055–1.643)	0.015
Marital status			
Single	1		
Married	1.975	(1.388–2.809)	< 0.001
Widowed/divorced	1.699	(1.162–2.485)	0.006
History of cigarette smoking			
No	1		
Yes	0.630	(0.558–0.710)	< 0.001
Employment status			
Unemployed	1		
Employed	0.940	(0.833–1.061)	0.317
Physical activity			
Light	1		
Moderate	0.965	(0.844–1.103)	0.598
High	0.802	(0.699–0.919)	0.002
Intensive	0.720	(0.627–0.827)	< 0.001

The logistic regression model for men aged 40 to 70 years old showed that being overweight was less likely in those with higher SES than in those with a low SES. Other factors involved in overweight men were history of cigarette smoking (OR = 1.495; *p* = 0.02) and moderate physical activity (OR = 1.355; *p* = 0.007) (Table 4).

**Table 4:** Regression model indicating factors associated with overweight in men

Variables retained in model	Odd’s Ratio (OR)	95% Confidence Interval for OR	*p*-value
SES			
Low	1		
Lower-middle	0.587	0.463–0.744	< 001
Upper-middle	0.659	0.522–0.832	< 001
High	0.742	0.585–0.941	< 001
History of cigarette smoking			
No	1		
Yes	1.495	(1.067–2.095)	0.02
Physical activity			
Light	1		
Moderate	1.355	1.087–1.688	0.007
High	1.196	0.980–1.461	0.078
Intensive	1.009	0.829–1.228	0.926

The result of the logistic regression analysis showed that being overweight was more likely among women with high SES (OR = 1.616; *p* <  0.001), women with an educational level below diploma or primary school (*p* <  0.001), and married (OR = 2.396; *p* <  0.001) or widowed women (OR = 2.109; *p* <  0.001). Moreover, women with a higher calorie intake were more likely to be overweight (OR = 1.000; *p* = 0.018). It was also revealed that being overweight is less likely in employed women than unemployed (OR = 0.846; *p* = 0.030), also less likely in women with a history of cigarette smoking (OR = 0.648; *p* = 0.012), and women with a high (OR: 0.718; *p* = 0.001) or intensive (OR = 0.730; *p* = 0.008) level of physical activity (Table 5).

**Table 5:** Regression model indicating factors associated with overweight in women

Variables retained in model	Odd’s Ratio (OR)	95% Confidence Interval for OR	*p*-value
SES			
Low	1		
Lower-middle	1.085	(0.926–1.272)	0.313
Upper-middle	1.187	(0.996–1.414)	0.056
High	1.616	(1.271–2.056)	< 0.001
Education level			
Illiterate	1		
Up to primary school	1.505	(1.307–1.732)	< 0.001
Under diploma	1.567	(1.251–1.962)	< 0.001
University degree	1.570	(0.995–2.392)	0.071
Employment status			
Unemployed	1		
Employed	0.846	(0.727–0.984)	0.030
Marital status			
Single	1		
Married	2.396	(1.634–3.516)	< 0.001
Widowed/divorced	2.109	(1.400–3.177)	< 0.001
History of cigarette smoking			
No	1		
Yes	0.648	(0.461–0.909)	0.012
Physical activity			
Light	1		
Moderate	0.864	(0.717–1.042)	0.125
High	0.718	(0.595–0.865)	0.001
Intensive	0.730	(0.578–0.922)	0.008
Calorie intake	1.000	(1.000–1.000)	0.018

## Discussion

This study investigated gender differences in determinants of being overweight among the 40- to 70-year-old population in Kharameh, located in southwestern Iran. About half of the study population was overweight. Being overweight was more prevalent among women than men (62.7% versus 43.6%), which is in line with previous studies [6, 9, 12, 13].

Gender, SES, marital status, educational level, smoking, and level of physical activity were found to be determinant factors of being overweight in the population under study. Female gender was a predictor for being overweight in the studied population [6, 9, 12, 13]; therefore, a gender-based analysis was also reported. In addition, being overweight was more likely among individuals with upper-middle and high SES. Khabazkhoob et al. (2017) confirmed that higher SES was significantly correlated with being overweight in the middle-aged population [9]. Probable reasons might be the prevalence of diseases such as hypothyroidism or diabetes mellitus in such age groups [23, 24] and the use of certain drugs and their metabolic side effects which may result in abnormal BMI [25]. Moreover, consistent with previous findings [1, 6] being overweight could be due to less attention paid to being in shape after marriage [26], as it was more likely to be overweight in married and widowed/divorced people. Individuals with any level of education were also at more risk of being overweight compared with those illiterate. As the educational level of the majority of the participants was illiterate or under diploma, the results about the relationship between educational level and being overweight should be interpreted with caution; the number of individuals with a university degree, whose nutritional literacy is expected to be significantly higher, was not enough for comparison. Clearly, the association between obesity and level of education is complex [27], and the following gender-based findings provide a clearer picture of the relationship. However, those with a history of cigarette smoking were less likely to be overweight, which might be a consequence of the role of smoking in suppressing appetite [28]. People with high and intensive physical activity were also less likely to be overweight. In line with the current findings, Bradbury et al. (2017) concluded that more intensive physical activity was associated with lower body fat percentage [29].

In women, the current study found SES, level of physical activity, employment status, educational level, marital status, and calorie intake to be determinant factors of being overweight. Women with a high SES were more likely to be overweight. Similar to these findings, Khabazkhoob et al. (2017) found that higher SES was significantly correlated with being overweight and obese [9]. Gouda et al. (2014) also confirmed that non-poor women were about 2 to 3 times more at risk of being overweight [30]. Moreover, women with high and intensive levels of physical activity were less likely to be overweight. Similarly, prior research identified low level of physical activity as a significant risk factor for being overweight [15, 16]. Numerous scientific studies have explained it in the perspective of the nutritional transition in developing countries or the shift to Western diets with highly-saturated fats, sugar, and refined and processed foods in addition to an increased prevalence of physical inactivity [30, 31]. The current study also revealed that being overweight was less prevalent among employed women. In line with these findings, Noh et al. showed that employed women had lower BMIs, although they also showed that employment status had various impacts on BMI by gender [32]. Sarma et al. found that unemployed women were at a 1.44-times higher risk of being overweight or obese than employed women [12]. In fact, unemployment can be associated with behavioral changes which affect diet with increased consumption of unhealthy foods and less physical activity that can lead to weight gain [32, 33]. In addition, women with an educational level below high-school diploma were at a higher risk of being overweight. Previous studies have also confirmed that lower education is related with being overweight [12]. It could be secondary to a lower likelihood of women without a university degree to find a job, which can result in a more sedentary lifestyle. Being married or widowed was another predictor for being overweight in women. This is consistent with other studies that have mentioned marriage as a predictor for being obese [1, 6] and overweight among women [17]. Prior studies have shown that entering marriage is associated with gaining weight [34]. The reason could be relaxing about being in shape after marriage [26]. Consistent with previous findings, higher calorie intake was another predictor for being overweight. In fact, in the face of high-caloric foods, more self-reported impulsivity is reported, which increases susceptibility to speeded detection of such foods in obese individuals [35]. Similar to previous studies [17, 28], the current research found that women with a history of cigarette smoking were less likely to be overweight. One reason for this association might be the role of smoking in suppressing appetite [28].

Among men, low SES, history of cigarette smoking and moderate physical activity were associated with being overweight. Men with lower SES were more likely to be overweight [13, 14], which was consistent with the findings in men. However, findings about the associations between SES and BMI are still controversial [9, 13, 14], which could be the result of variations in the study population’s age group, nutritional habits, or level of daily activity. Among lifestyle variables, history of cigarette smoking and physical activity were significant predictors of being overweight among men. The current study showed that male smokers were more likely to be overweight. A probable reason could be having been a smoker in previous years and not currently a smoker. In line with this logic, Dare et al. (2015) confirmed that former smokers were more likely to be obese compared with current and never smokers [36]. Among men, only moderate physical activity was in significant association with being overweight. Therefore, men with moderate physical activity were at higher risk of being overweight. However, a moderate level of physical activity cannot be as effective as intense activity on an individual’s BMI. Moreover, given that they have been physically active, they cared less about calorie intake, which resulted in an abnormal BMI. In addition, considering the age of the studied sample, this might be due to the prevalence of diseases such as hypothyroidism or diabetes mellitus [23, 24] or the use of certain drugs and their metabolic side effects which can result in an abnormal BMI [25].

### Limitation and strength

One of the main limitations of this study was a lack of information regarding the nutritional habits of the participants, although it could be accompanied with a remarkable recall bias. Another limitation was having no information about the participants’ medical and medication history, because some diseases and/or medications have proven associations with increased weight. Furthermore, we studied determinant factors of being overweight but not obesity. However, this study is unique in that the census method was used and 10,663 individuals aged between 40 and 70 years were recruited during 2015 and 2016. It is worth noting that physical activity was measured through MET, which is one of the most reliable measures for level of physical activity.

## Conclusions

The current study revealed differences in socio-demographic and lifestyle factors of being overweight according to gender. As being overweight was more prevalent among women, the priority of policies to control this issue should be focused on women. For further actions to control future obesity, considering related factors for each gender separately should not be neglected.

## Data Availability

The datasets used and analyzed during the current study are available by sending an email to the owner of data (Abbas Rezaianzadeh; rezaiana@gmail.com).

## Abbreviations

BMI: High Body Mass Index
WHO: World Health Organization
SES: Socio-economic status
PERSIAN: Prospective Epidemiological Research Studies in Iran
MET: Metabolic equivalent rate
SPSS: Statistical Package for Social Sciences
OR: Odds ratio
CI: Confidence interval
